# Glucose-Regulated Protein 78 Autoantibodies Are Associated with Carotid Atherosclerosis in Chronic Obstructive Pulmonary Disease Patients

**DOI:** 10.4049/immunohorizons.1900098

**Published:** 2020-02-21

**Authors:** Thi K. Tran-Nguyen, Divay Chandra, Kaiyu Yuan, Phani K. Patibandla, Khanh T. Nguyen, Palaniappan Sethu, Yingze Zhang, Jianmin Xue, James A. Mobley, Young-il Kim, Ali Shoushtari, Joseph K. Leader, Jessica Bon, Frank C. Sciurba, Steven R. Duncan

**Affiliations:** * Department of Medicine, University of Alabama at Birmingham, Birmingham, AL 35294;; † Department of Medicine, University of Pittsburgh, Pittsburgh, PA 15213;; ‡ Department of Radiology, University of Pittsburgh, Pittsburgh, PA 15213;; § Department of Medicine, VA Pittsburgh Healthcare System, Pittsburgh, PA 15213

## Abstract

Atherosclerosis prevalence is increased in chronic obstructive pulmonary disease (COPD) patients, independent of other risk factors. The etiology of the excess vascular disease in COPD is unknown, although it is presumably related to an underlying (if cryptic) systemic immune response. Autoantibodies with specificity for glucose-regulated protein 78 (GRP78), a multifunctional component of the unfolded protein response, are common in COPD patients and linked to comorbidities of this lung disease. We hypothesized anti-GRP78 autoreactivity might also be a risk factor for atherosclerosis in COPD patients. Carotid intima-medial thickness (cIMT) was measured in 144 current and former smokers by ultrasound. Concentrations of circulating IgG autoantibodies against full-length GRP78, determined by ELISA, were greater among subjects with abnormally increased cIMT (*p* <, 0.01). Plasma levels of autoantibodies against a singular GRP78 peptide segment, amino acids 246–260 (anti-GRP78_aa 246–260_), were even more highly correlated with cIMT, especially among males with greater than or equal to moderate COPD (*r*_s_ = 0.62, *p* = 0.001). Anti-GRP78_aa 246–260_ concentrations were independent of CRP, IL-6, and TNF-α levels. GRP78 autoantigen expression was upregulated among human aortic endothelial cells (HAECs) stressed by incubation with tunicamycin (an unfolded protein response inducer) or exposure to culture media flow disturbances. Autoantibodies against GRP78_aa 246–260_, isolated from patient plasma by immunoprecipitation, induced HAEC production of proatherosclerotic mediators, including IL-8. In conclusion, anti-GRP78 autoantibodies are highly associated with carotid atherosclerosis in COPD patients and exert atherogenic effects on HAECs. These data implicate Ag-specific autoimmunity in the pathogenesis of atherosclerosis among COPD patients and raise possibilities that directed autoantibody reduction might ameliorate vascular disease in this high-risk population.

## INTRODUCTION

Chronic obstructive pulmonary disease (COPD) is the fourth leading cause of death in the United Stated (1). Although the respiratory abnormalities of COPD result in considerable morbidity and mortality, the majority of deaths among these patients are due to atherosclerotic cardiovascular disease (CVD) (2–5). The increased prevalence of atherosclerosis in COPD is not fully explained by smoking exposure, age, hypertension, and other established CVD risks (6, 7). Chronic inflammation has been implicated in the development of atherosclerosis in COPD patients, although the nature of the causal immunological mechanism(s) remains unknown (4, 8).

We, and others, have shown that autoimmunity can mediate chronic inflammation in COPD (9–11). IgG autoantibodies with diverse specificities and effects are frequently present and associated with immune complex deposition and complement activation in the diseased lungs of COPD patients (9–12). B cell aggregates in nonlymphoid organs are a pathognomonic feature of chronic immune responses that exert numerous pathological effects, in addition to their production of Abs (13), and these are also a common finding in COPD lungs (14). B cells infiltrate into diseased tissues because of the local production of chemotactic factor C-X-C chemokine 13 (CXCL13), and this trafficking mediator is overexpressed in COPD lungs (15). Circulating concentrations of BAFF (also known as BLyS), a trophic factor for B cell survival and function, are also increased proportionately to disease severity among patients with conventional autoimmune syndromes as well as those with COPD (16).

IgG autoantibodies with avidity for glucose-regulated protein 78 (GRP78) are present in many COPD patients (12). GRP78 is a multifunctional endoplasmic reticulum (ER) stress and heat shock protein (HSP) that plays an important role in the unfolded protein response (UPR) (17, 18). Pulmonary GRP78 production is increased by various stresses, and high levels of this protein are expressed in COPD lungs (12, 19). In addition to its intracellular localization and functions, GRP78 can also be exported into extra-cellular spaces, whereupon it acts as a cytokine by cognate binding to specific cell surface receptors that transduce danger signals (18). Anti-GRP78 Abs can cross-link and enhance the signaling events mediated by these GRP78-receptor complexes, resulting in significant alterations of several diverse cellular functions (12, 20–22).

Because anti-GRP78 autoantibodies are common in COPD patients, and have myriad potentially injurious effects (12, 20–22), we hypothesized that the “spillover” of these autoantibodies from their putative origin in injured lungs (14, 15) might also be involved in vascular pathogenesis. We tested our hypothesis by examining the association in COPD patients of circulating anti-GRP78 autoantibodies with carotid intima-medial thickness (cIMT), a facile correlate of systemic atherosclerotic disease (23). Additional studies looked for expression of GRP78 among human aortic endothelial cells (HAECs), surveyed for differences of epitope(s) specificity among the autoantibodies most highly associated with cIMT abnormalities, and sought to determine if patient-derived anti-GRP78 autoantibodies can deleteriously alter HAEC functions.

## MATERIALS AND METHODS

### Study participants

Subjects were enrolled from the Pittsburgh COPD Specialized Center for Clinically Oriented Research cohort. This population consists of 40- to 79-y-olds with minimum 10 pack-year smoking histories and excluded participants with other significant lung diseases, vascular or cardiac events in the preceding year, prior thoracic surgery, or a body mass index >35 kg/m^2^. Each subject completed demographic and medical history questionnaires, pulmonary function tests (PFT), chest computed tomography (CT) examinations, carotid ultrasounds, and blood sample collection. Written informed consent was obtained from each participant.

This study was approved by the Institutional Review Boards at the University of Pittsburgh and University of Alabama at Birmingham.

### Lung assessments

Results of pulmonary function tests were adjusted to standard population-derived predicted values (24). COPD was defined by the presence of fixed expiratory airflow obstruction, (i.e., a ratio of the forced expiratory volume in 1 s [FEV1] to forced vital capacity [FVC] [FEV1/FVC] <0.70) (24). Subjects with COPD were further classified by severity according to Global Initiative for Chronic Obstructive Lung Disease (GOLD) criteria, which are based on percent predicted (pp) FEV1: GOLD 1 = FEV1pp >80%; GOLD 2 = >50% FEV1pp <80%; GOLD 3 = >30% FEV1pp <50%; and GOLD 4 = FEV1pp <30% (24).

Chest CT scans were performed with a LightSpeed VCT 64-Detector Scanner (GE Healthcare, Little Chalfont, United Kingdom) without i.v. contrast and quantified as detailed previously (25). Briefly, the extent of emphysema (defined as parenchymal lung loss) was calculated by traditional density mask analyses on inspiratory CT scans as the percentages of total lung volume with attenuation ≤−2950 Hounsfield units (F_950_). The presence of emphysema was defined as F_950_ >0.05 (26).

### Carotid ultrasounds

Carotid artery ultrasound studies were performed in consecutively recruited subjects using a GE 9L Linear Array Transducer with a frequency of 3–10 MHz (GE Healthcare, Tokyo, Japan) and GE Vivid 7 Dimension Ultrasound (GE Vingmed Ultrasound, Horten, Norway). Intima-media thickness was measured using automated edge detection software that located the lumen-intima and media-adventitia echo boundaries at subpixel resolution and made >50 measurement of the intima-media thickness along a 1-cm segment of the distal common carotid artery 1 cm proximal from the carotid bifurcation at end-diastole. These >50 measurements were averaged to calculate the intima-media thickness for each side, and the mean of the values from the left and right carotid was used for data analyses. cIMT ≥0.9 mm were classified as abnormal.

### Autoantigen discovery

The nonbiased methods that resulted in discovery of GRP78 autoimmunity in COPD have been previously detailed (12).

### Anti-GRP78 autoantibody assays

Full-length human GRP78 was cloned into the pDB-HisGST vector (DNASU plasmid repository, Arizona State University, Tempe, AZ) containing the N-terminal 6*His-tag. The protein was overexpressed in *Escherichia coli* strain BL21(DE3) (Thermo Fisher Scientific, Waltham, MA) that was propagated in Luria-Bertani medium. After lysis and centrifugation, the recombinant GRP78 (rGRP78) was purified on nickel columns.

rGRP78 was used as the substrate of ELISA by dilution to 0.5 μg/ml in PBS prior to incubation in ELISA plate wells (100 μl/well) overnight at 4°C. After thorough washing and blocking, plasma specimens were added to the wells at 1:100 dilutions and incubated for 2 h. Following additional washes, alkaline phosphatase (AP)–conjugated goat anti-human Ab (Invitrogen, Carlsbad, CA) at 1:5000 dilution was added and color developed with a pNPP substrate system (Kirkegaard & Perry Lab, Gaithersburg, MD). ODs were determined at 405 nm.

### Inflammatory mediators

High-sensitivity CRP (hs-CRP) was measured in plasma samples using an ultrasensitive electrochemiluminescence immunoassay according to the manufacture’s protocol (Meso Scale Discovery, Gaithersburg, MD).

IL-6 and TNF-α levels were measured using ELISA kits according to the manufacturer’s instructions (R&D Systems, Minneapolis, MN).

### Cardiovascular risk covariates

Hypertension was defined as systolic blood pressure >140 mm Hg measured during the study visit or use of an antihypertensive medication. Hyperlipidemia and diabetes were defined by the use of a lipid-lowering or diabetic medication, respectively, or patient reports of these diagnoses. Family histories of vascular disease were established by patient report.

### HAECs

HAECs were purchased (Invitrogen) and expanded in Medium 200 with low-serum growth supplement from the same supplier, 5% FBS, and 1% penicillin-streptomycin. Standard plastic tissue culture flasks were coated with 5 μg/ml fibronectin (Sigma-Aldrich, St. Louis, MO) for 2 h at 37°C, before seeding with HAECs. HAECs were detached from cultures at ~70% con-fluency with 0.05% trypsin/0.53 mM EDTA for subsequent plating.

### Microfluidic endothelial cell culture model

HAECs were cultured within the endothelial cell culture model (ECCM) and subjected to either normal pulsatile flow (as seen in linear arterial sections) or oscillatory-disturbed flow associated with atherosclerosis-susceptible regions (e.g., arterial bifurcations) using cell culture chambers and flow loops that have been previously detailed (27). After culture within the ECCM for 24 h, the HAECs were washed with cold PBS, then fixed on the polymeric membranes with 4% paraformaldehyde for immunofluorescence imaging (27).

### Immunofluorescence staining and confocal microscopy

Fixed HAECs on ECCM polymeric membranes were washed with PBS, blocked with 5% BSA at room temperature for 30 min, and incubated overnight at 4°C with anti-GRP78 Ab (cat# sc-1051; Santa Cruz Biotechnology, Dallas, TX) or normal goat IgG as isotype control (cat# sc-2020; Santa Cruz Biotechnology), both of which were diluted 1:50 in 1% BSA in PBS. Membranes were again washed three times with PBS, 5 min each, prior to incubation with a 1:5000 dilution of anti-goat F(ab′)_2_ conjugated to Texas Red (cat# NB120–6523; Novus Biologicals, Centennial, CO). Stained membranes were air-dried before adding mounting solution with DAPI (Vector Laboratories, Burlingame, CA).

Images were captured using an A1R confocal microscope (Nikon Instruments, Melville, NY).

### Flow cytometry to assess GRP78 expression on HAECs

HAECs were treated with tunicamycin (2.5 μg/ml; Millipore-Sigma, Burlington, MA), an inducer ofER stress, orDMSO (solvent control), also at 2.5 μg/ml, in Medium 200 for 48 h. Cells were collected by scraping and then washed one time with FACS buffer (HBSS + 0.1% BSA + 0.1% sodium azide) before being incubated with 0.5 μg in 50 μl of FACS buffer of either GRP78 rabbit polyclonal Ab (cat# 3183S; Cell Signaling Technology, Beverly, MA) or isotype control (cat# 02–61020; Thermo Fisher Scientific) for 25 min on ice. Cells were then washed one time before incubation with anti-rabbit IgG (H+L) F(ab′)_2_ fragments (PE-conjugated), at 1:500 dilution in FACS buffer for another 25 min. 7-AAD was added as the final step.

Data were acquired on a BD LSR-II Cytometer (BD Biosciences, San Jose, CA) and analyzed by FlowJo software (FlowJo, Ashland, OR).

### GRP78 peptides

Potential linear epitopes of GRP78 (8–20 aa in length) were identified in silico using the BepiPhred-2.0 sequential B cell-epitope predictor (http://www.cbs.dtu.dk/services/BepiPred/). The corresponding peptides were synthesized by Thermo Fisher Scientific and are listed in Supplemental Table I. Each peptide was biotinylated at the N terminus, after addition of an aminohexanoic (Ahx) linker, to minimize steric hindrance. The identity and purity of these synthesized peptides were confirmed by mass spectrometry.

For use in ELISA, the biotinylated peptides were diluted to 0.5 μg/ml in PBS and added to wells of a streptavidin-coated microplate (R&D Systems) for overnight incubation at 4°C. Subsequent steps were identical to those described for full-length rGRP78 ELISA (above).

### Isolation of anti-GRP78_aa 246–260_ autoantibodies

IgG in pooled plasma from COPD patients was precipitated by ammonium sulfate, resuspended, dialyzed, and then captured on protein G columns (Life Technologies, Carlsbad, CA) (12). After several column-volume washes, the IgG was eluted with 100 mM glycine (pH 3.0) and the eluant pH neutralized with Tris buffer. IgG in this solution was concentrated using 50-kDa centrifugal filters and measured by spectrophotometer. Control IgG was obtained by identical isolations from pooled normal plasma specimens. The IgG identity of both preparations was confirmed by SDS gel electrophoresis.

Autoantibodies with specificity for GRP78_aa 246–260_ were isolated from the COPD IgG by immunoprecipitation. Synthesized, biotinylated GRP78_aa 246–260_ peptide was coupled to streptavidin agarose resin columns, following the manufacturer’s instructions (Thermo Fisher Scientific). The columns were repeatedly washed with PBS and then incubated with the COPD IgG overnight at 4°C. After another exhaustive washing, anti-GRP78_aa 246–260_ autoantibody was eluted with acidified buffer, neutralized, and concentrated using centrifugal filters. The purity and avidity of anti-GRP78_aa 246–260_ autoantibody were confirmed by SDS gel electrophoresis and Western blots against denatured rGRP78. The Ab was diluted with complete media and sterile-filtered before incubation with HAECs. Endotoxin concentrations in these autoantibody preparations were below the detection threshold of a Pierce LAL Chromogenic Endotoxin Quantitation Kit (Pierce Biotechnology, Rockford, IL).

IgG purified by protein G from pooled normal human serum (cat# I4506; Sigma-Aldrich) was used as the control for the GRP78_aa 246–260_. This control IgG preparation was also subjected to the same incubations with agarose resin columns, washes, elutions, and endotoxin assays as the GRP78_aa 246–260_ IgG.

### HAEC treatment with anti-GRP78_aa 246–260_ autoantibodies

HAECs (2 × 10^5^/ml) were seeded in 12 well-plates that had been previously coated with fibronectin (5 μg/ml). After 2 d of culture, the HAECs were incubated with either anti-GRP78_aa 246–260_ IgG or control IgG (each at 5 μg/ml) for 8 h before harvesting for RNA extraction. Supernatants were collected from otherwise identically treated HAEC culture after 24 h for ELISA assays.

### RNA isolation and NanoString analyses

Harvested HAECs were washed and lysed, and RNA was isolated using the reagents and procedures supplied in a kit (RNAeasy Plus; Qiagen, Hilden, Germany). RNA quality was confirmed, and concentrations were determined by nanodrop.

The RNA was cleaned and concentrated using a kit (Zymo Research, Irvine, CA) and eluted in RNase-free water. One hundred nanograms was added to the Reporter CodeSet and Capture ProbeSet of the premade Human Inflammation Panel (NanoString Technologies, Seattle, WA) and hybridized overnight at 65°C, per the manufacturer’s protocol. Samples were subsequently processed on a NanoString Technologies Prep station and Digital Analyzer, and the data were analyzed with this company’s nSolver 4.0 Software. Volcano plots were generated by using R version 3.5.0. with packages ggplot2, dplyr, and ggrepel.

### Quantitative real-time RT-PCR

cDNA from the HAEC RNA aliquots were synthesized using SuperScript IV First-Strand Synthesis System (Invitrogen). Quantitative real-time RT-PCR, used to confirm selected NanoString findings, was performed on this cDNA using iQTM SYBER Green Supermix (Bio-Rad Laboratories, Hercules, CA) according to the manufacturer’s recommendation. The primers used in these reactions were produced by Integrated DNA Technologies (Skokie, IL) and included IL-8 forward (5′-GACCACACTGCGC CAACAC-3′) and reverse (5′-CTTCTCCA CAACCCTCTGCAC-3′) and IFN regulatory factor-7 (IRF-7) forward (5′-ACCCTGG CTGTGCCGAGT-3′) and reverse (5′-AAGCACTCGATGTCGT CATAGAG-3′). GAPDH primers were purchased from Bio-Rad Laboratories. Reactions were performed in triplicate and normalized to GAPDH using the comparative 2^−ΔΔCT^ method (28).

### ELISA for IL-8

HAECs culture supernatants collected at 24 h after IgG stimulation were used to measure IL-8 concentrations by ELISA (R&D Systems). Standard curves and concentrations were calculated by using the 4PL curve-fit method with online software (https://mycurvefit.com/).

### Statistical analyses

Continuous or ordered variables were compared using the Kruskal–Wallis test. Dichotomous variables were compared by χ^2^. Spearman rank-order correlations were used to identify associations between continuous or ordinal variables. Multivariable logistic regression models were constructed to identify variables, which predict abnormal cIMT, and to control various covariates and potential confounders. The Wald test was used to identify variables that significantly explain the abnormal cIMT outcome.

Analyses were performed using Prism software, version 5.0b. Summary statistics are reported as medians, ranges or means ± SD for continuous variables, and proportions (%) for categorical variables. Statistical significance was defined as two-tailed *p* < 0.05.

## RESULTS

### Subjects

The study cohort was composed of 144 consecutive Specialized Center for Clinically Oriented Research participants who had both anti-GRP78 IgG measurements and carotid ultrasounds. Eighty-one of these subjects had normal spirometry, despite extensive smoking histories (smoke controls [SC]), and 63 had COPD. There were no attempts to select participants based on gender, or other demographic or clinical features, but fortuitously equal numbers of males and females were recruited (e.g., *n* = 72). Clinical and demographic characteristics of these populations are delineated in Table I.

### Autoantibodies against full-length GRP78

**FIGURE 1.**
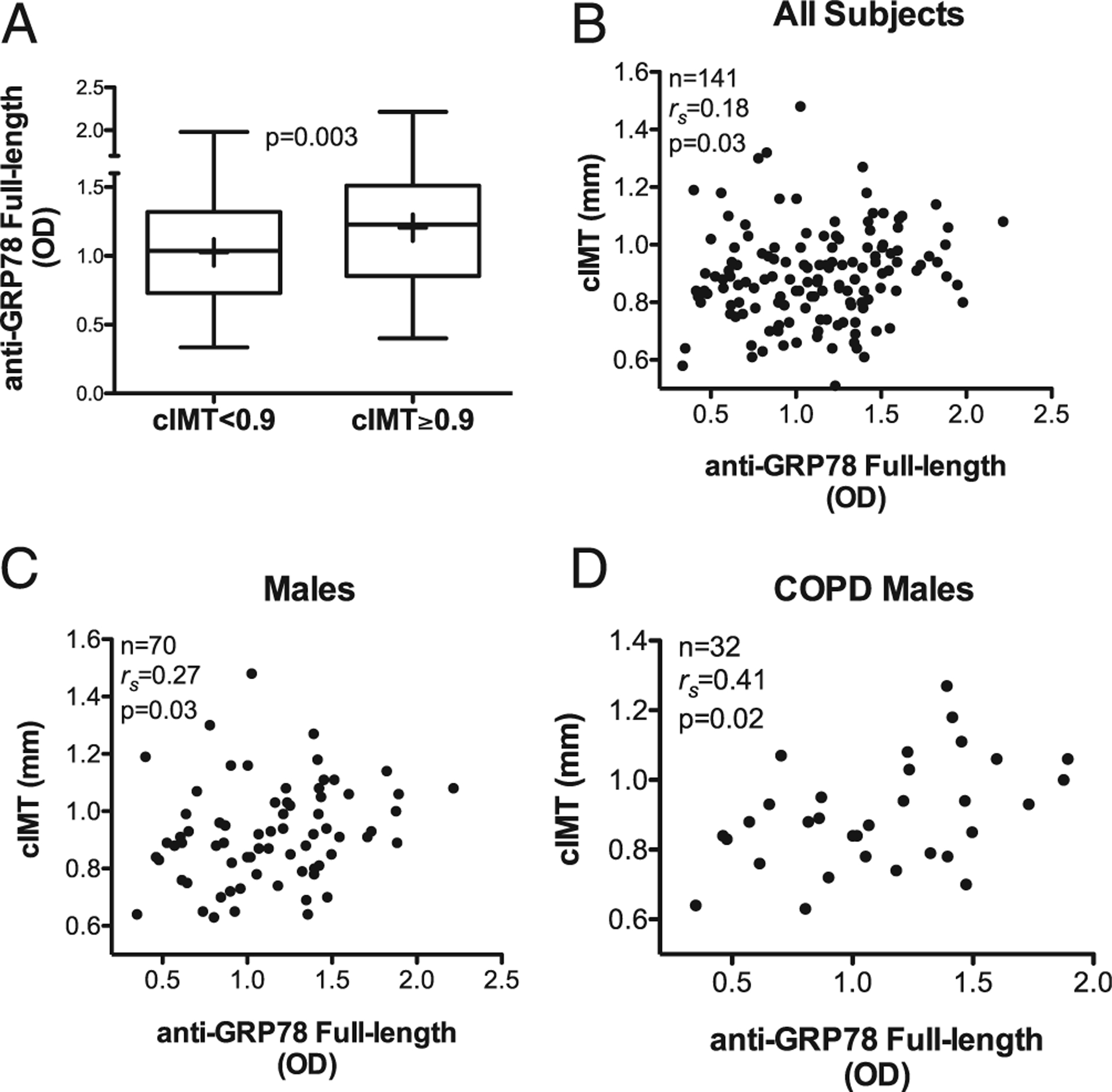
Autoantibodies to full-length GRP78 protein in the study cohort were associated with carotid atherosclerosis. (**A**) Anti-GRP78 autoantibody levels (as ODs measured by ELISA) were greater in the subpopulation with abnormal cIMT, defined by cIMT ≥0.9 mm, compared with those with normal cIMT values. The lowest, second lowest, middle, second highest, and highest lines represent the 10th, 25th, median, 75th, and 90th percentiles, respectively. Means are denoted by the plus sign (+). (**B**) Correlation between the ELISA OD for autoantibodies with specificities to full-length GRP78 and cIMT in the aggregate study population. (**C**) Correlation between the levels of autoantibodies to full-length GRP78 and cIMT in male subjects. (**D**) Correlation between the levels of autoantibodies to full-length GRP78 and cIMT in males with COPD.

Levels of circulating IgG autoantibodies to full-length rGRP78 did not differ between COPD and SC (Table I). However, autoantibody concentrations were significantly greater among the 64 subjects with carotid artery disease, defined as cIMT ≥0.9 mm, in comparison with the 80 subjects with normal cIMT (1.2 ± 0.4 versus 1.0 ± 0.4 OD units, *p* = 0.003) (Fig. 1A) and were weakly correlated with their cIMT values (Fig. 1B).

Although anti-GRP78 autoantibody levels were near identical in both male and female participants (1.1 ± 0.4 OD units in each), the clinical phenotype associations with anti-GRP78autoreactivity have been previously reported to be gender biased (12). Accordingly, we performed post hoc analyses and found the association of anti-GRP78 to cIMT was greatest among male subjects (Fig. 1C), whereas this relationship was NS among females (*r*_s_ = 0.08, *p* = 0.52). This autoantibody-cIMT association was stronger still in the males with COPD (Fig. 1D), compared with the COPD females (*r*_s_ = −0.02, *p* = 0.93).

### Autoantibodies against GRP78_aa 246–260_

To determine if one or more GRP78 autoantibody epitopes were singularly associated with cIMT abnormalities, we conducted a series of ELISA using linear, internal GRP78 peptides as the plate-bound ligands. Autoantibodies with avidities for one of these peptides, a 15-mer (ATNGDTHLGGEDFDQ), corresponding to GRP78 amino acid 246–260 (https://www.uniprot.org/uniprot/P11021), had the greatest association with cIMT within the aggregate study population (*r*_s_ = 0.23, *p* < 0.01) compared with results with the other peptide ligands (Supplemental Table II) or with full-length rGRP78 (Fig. 1B). The unique specificity of anti-GRP78_aa 246–260_ may also be substantiated by finding a poor correlation between levels of this autoantibody and the anti–full-length rGRP78 (*r*_*s*_ = 0.13, *p* = 0.13).

**FIGURE 2.**
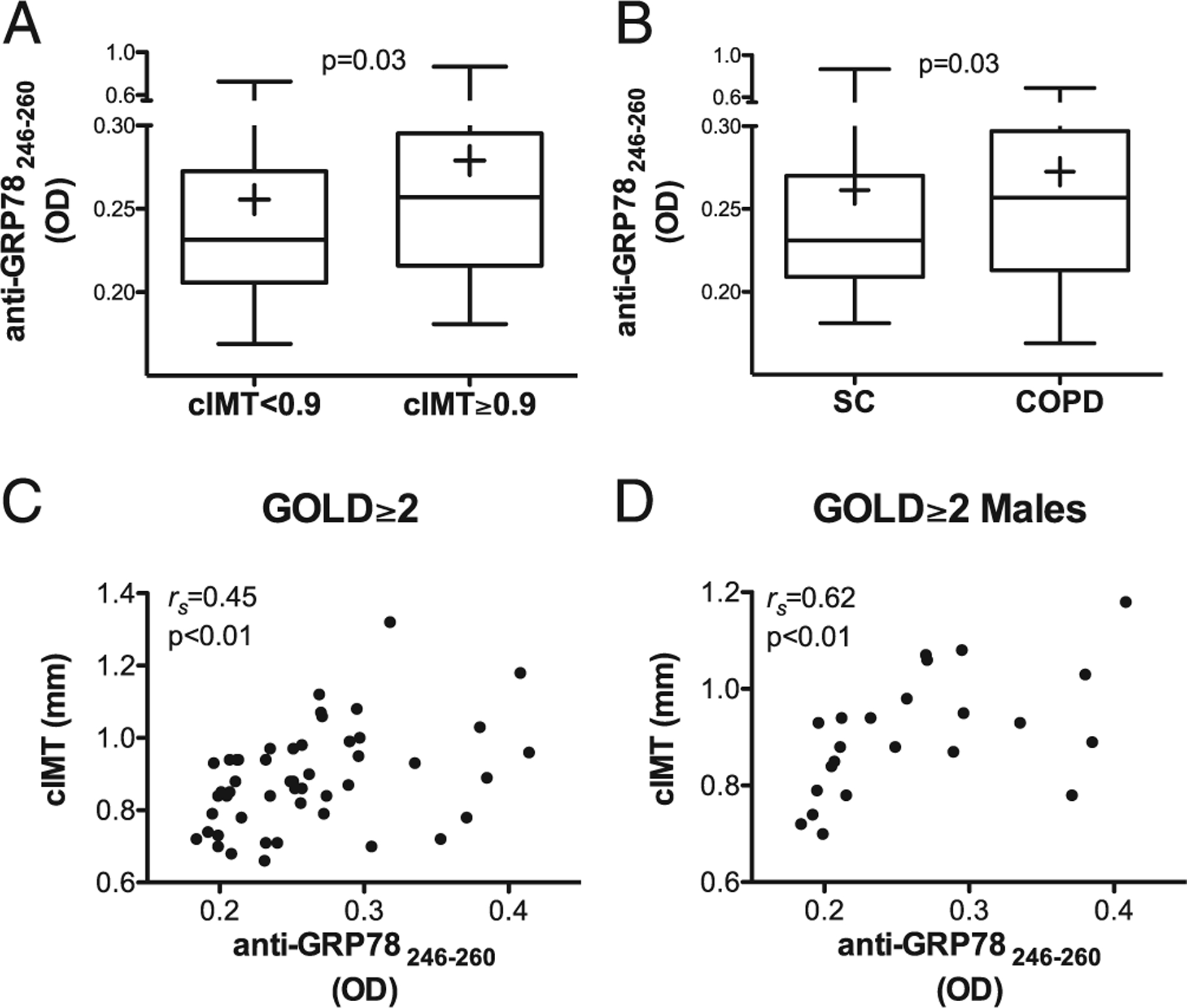
Autoantibodies to GRP78_aa 246–260_ peptide were even more related to measures of carotid artery disease. (**A**) Circulating IgG autoantibodies to GRP78_aa 246–260_ peptide (as OD measured by ELISA) were higher in the population with abnormal cIMT (≥0.9 mm), compared with those with normal cIMT. The lowest, second lowest, middle, second highest, and highest lines represent the 10th, 25th, median, 75th, and 90th percentiles, respectively. Means are denoted by the plus sign (+). (**B**) Anti-GRP78_aa 246–260_ autoantibodies were increased in COPD patients compared with SC. (**C**) Correlation between levels of anti-GRP78_aa 246–260_ autoantibodies and cIMT among patients with moderate to advanced COPD (GOLD ≥2) (24). (**D**) Correlation between anti-GRP78_aa 246–260_ autoantibodies and cIMT among males with COPD GOLD ≥2.

Circulating anti-GRP78_aa 246–260_ levels were near identical among males and females (0.27 ± 0.10 OD units each). Study participants with abnormal cIMT also had higher anti-GRP78_aa 246–260_ autoantibody concentrations compared with those with normal cIMT (Fig. 2A). Autoantibodies against this internal epitope were slightly, but significantly, greater among COPD patients compared with SC (Fig. 2B). The correlation between values for anti-GRP78_aa 246–260_ and cIMT was, again, strongest in the COPD patients, particularly among those with ≥GOLD 2 severity (Fig. 2C), and among the males within this disease subpopulation (Fig. 2D).

These autoantibody-cIMT associations appeared to be independent of nonspecific inflammatory mediators, given the absence of any correlations between anti-GRP78_aa 246–260_ autoantibody levels and hs-CRP, IL-6, or TNF-α (Supplemental Fig. 1).

**FIGURE 3.**
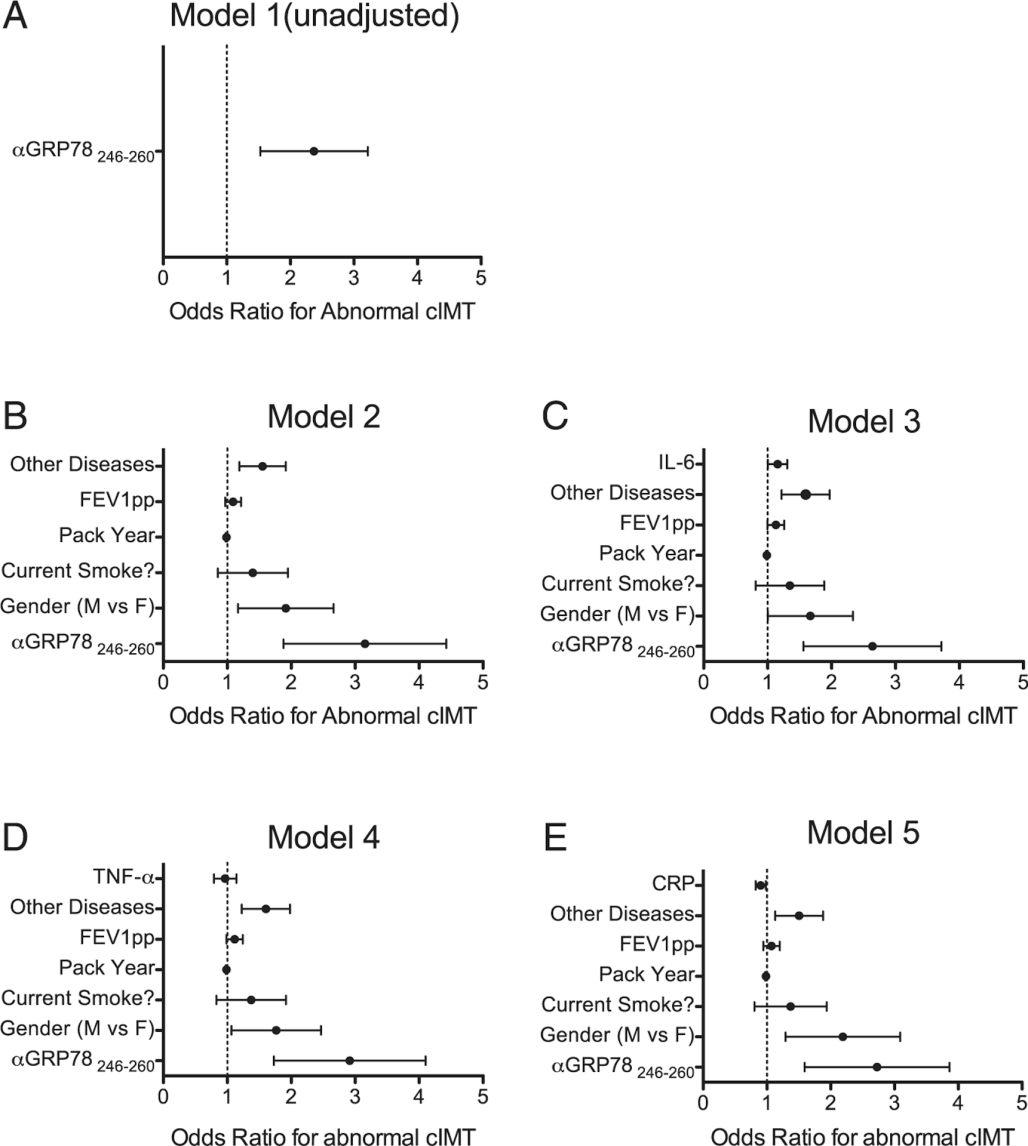
Logistic models to predict the relationships of anti-GRP78_aa 246–260_ autoantibodies with the presence of abnormal cIMT (≥0.9 mm). (**A**) Unadjusted model shows the OR of having an abnormal cIMT per an increase in 0.1 OD of anti-GRP78_aa 246–260_ autoantibodies. (**B**) Model 2 has been adjusted by gender, current smoking status, pack years, FEV1pp, and the presence of other chronic diseases associated with atherosclerosis (hypertension, diabetes, and hyperlipidemia). (**C**) Model 3 has been adjusted to include all the variables in model 2, plus the addition of circulating IL-6 (picograms per milliliter). (**D**) Model 4 has been adjusted to all the variables in model 2, with the addition of TNF-a (picograms per milliliter). (**E**) Model 5 has been adjusted to all the variables in model 2, with the addition of hs-CRP level (micrograms per milliliter). Bars denote 95% CI.

Logistic regression was used to model the relationship between anti-GRP78_aa 246–260_ autoantibody levels and the development of carotid atherosclerosis (defined as cIMT ≥0.9 mm). In the single independent variable (unadjusted) model, the odds ratio (OR) for an abnormal cIMT is 2.07 (95% confidence interval [CI] =1.08–3.96) per each increase of anti-GRP78_aa 246–260_ autoantibodies by 0.1 OD unit (Fig. 3A). The cIMT-autoantibody association remained significant (OR = 2.6, 95% CI = 1.3–5.6) after adjusting for potentially confounding subject characteristics and other CVD risk factors, such as gender, current smoking status, pack years, FEV1pp, and the presence of other chronic diseases associated with atherosclerosis (Fig. 3B). Similarly, the association between anti-GRP78_aa 246–260_ autoantibody levels and abnormal cIMT still remained significant after including other, albeit nonspecific, inflammatorymediatorssuchasIL-6,TNF-α, and hs-CRP independent variables in the logistic regression model (Fig. 3C–E).

### HAEC expression of GRP78

To establish that the GRP78 autoantigen is present among HAEC, and to explore conditions that might modify production of this autoantigen, we examined GRP78 expression in vascular cells exposed to various stresses.

**FIGURE 4.**
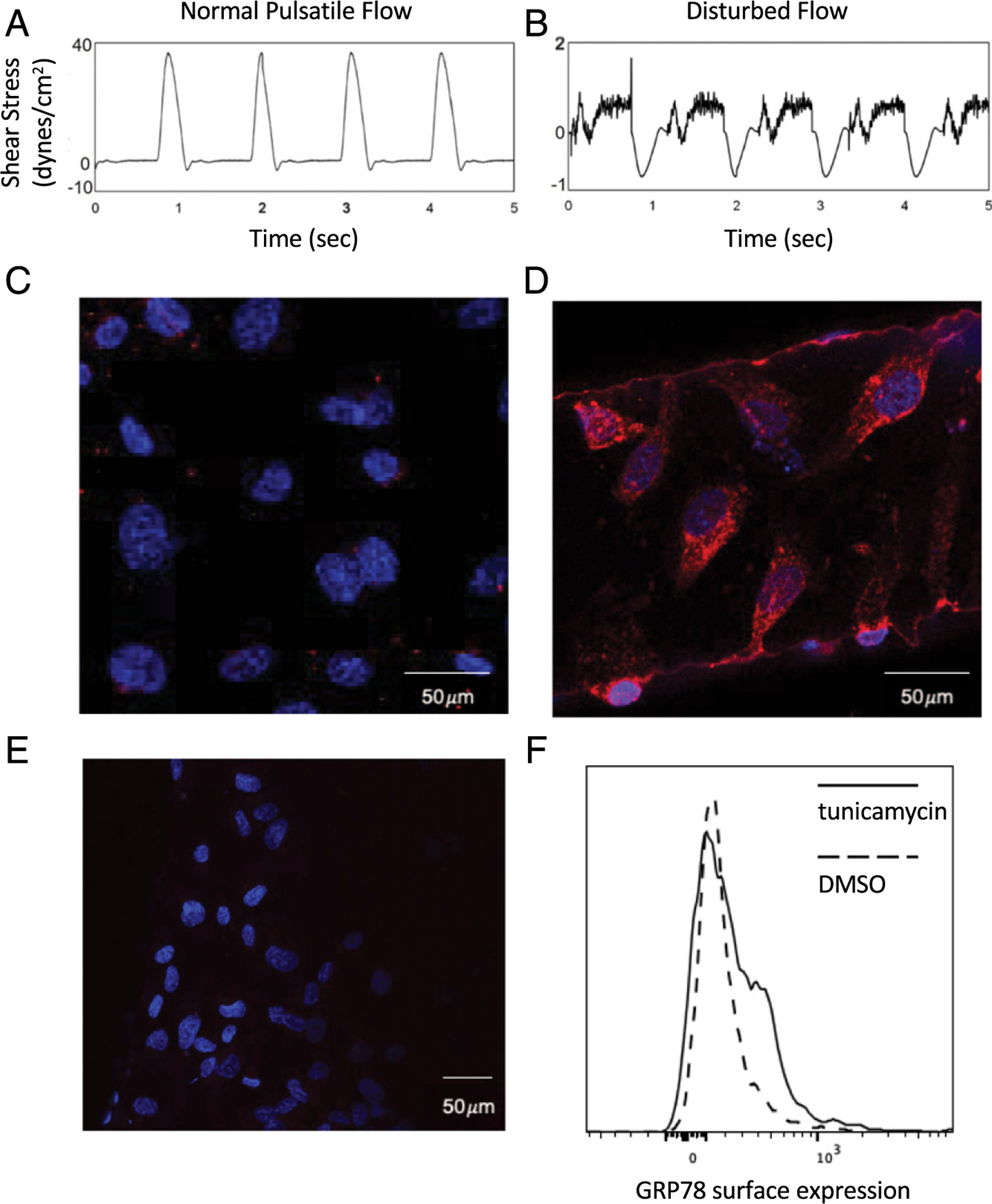
GRP78 expression on HAECs exposed to stresses of disturbed media flow and UPR induction. Shear stress patterns of normal pulsatile flow (**A**) or disturbed flow (**B**) generated inside the microfluidic ECCM chamber (27). Confocal microscopy showed that GRP78 (red stain) on HAECs grown under conditions of normal pulsatile flow was limited (**C**), in contrast to GRP78 expression on HAECs subjected to stressful conditions that mimic disturbed fluid flows (**D**) as seen in vascular niches where atherosclerotic plaques tend to develop. Blue stain denotes DAPI. (**E**) Normal goat IgG (isotype control) followed by the same secondary Abs as in (C) and (D) [anti-goat F(ab′)_2-_Texas Red] show the GRP78 staining of the latter is specific. (**F**) Tunicamycin (2.5 μg/ml) increased surface GRP78 expression on HAECs compared with DMSO (2.5 μg/ml) (carrier control) treatment.

A microfluidic device (27) was used to examine effects of altering culture media flow conditions (Fig. 4A, 4B) on GRP78 expression. Confocal microscopy showed that GRP78 expression was limited among HAECs cultured in conditions of normal pulsatile flow, whereas this autoantigen was significantly increased by stresses imposed by disturbed flow, as seen at arterial bifurcations (Fig. 4C–E) (27).

HAECs incubated 48 h with tunicamycin, an inducer of the UPR and ER stressor, also exhibited increased surface expression of GRP78, compared with cells treated with DMSO control (Fig. 4F).

### Potential pathogenic functions of anti-GRP78_aa 246–260_ autoantibodies

In addition to their widely known direct and indirect cytotoxic functions (13, 29, 30), IgG Abs can also evoke other, more specific, physiological effects, particularly if they bind to and cross-link cell surface receptors that transduce signals (12, 20–22, 31).

The potential functional effects of the anti-GRP78_aa 246–260_ autoantibodies that were most significantly related to cIMT in COPD patients (Fig. 2) were examined. We were especially interested in finding evidence that these autoantibodies directly exerted one or more clinically relevant pathological effects in HAECs.

**FIGURE 5.**
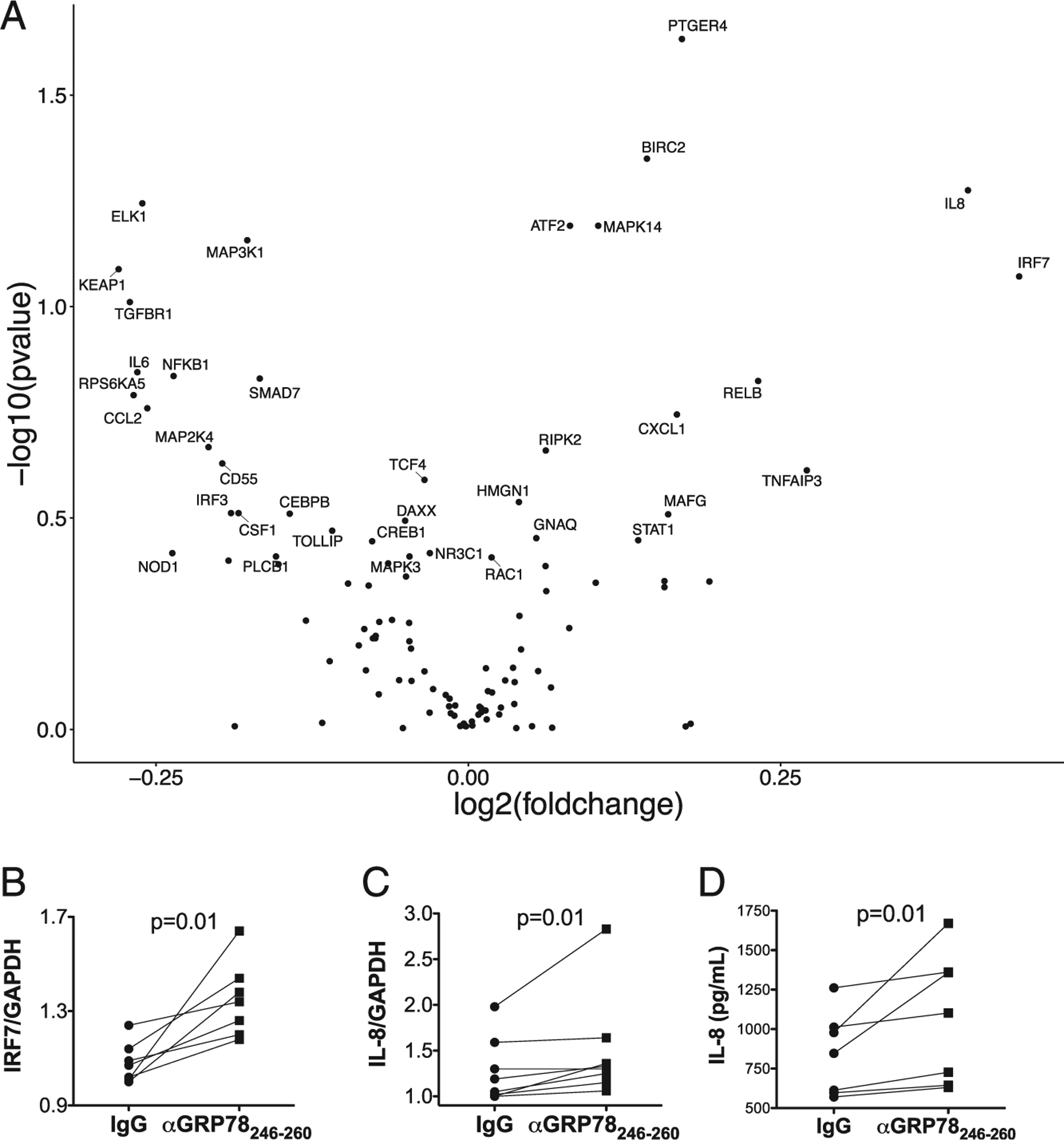
Anti-GRP78_aa 246–260_ autoantibodies altered HAEC gene expression. (**A**) Volcano plot shows fold changes (log values) in levels of selected HAECs gene expressions, measured by NanoString assay (NanoString Human Inflammatory Panel), versus significance (*p* values), in HAECs treated with anti-GRP78_aa 246–260_ autoantibodies isolated from patient plasma. Changes are relative to measures in concurrent HAECs treated with normal IgG (*n* = 4 pairs). Validations of NanosString assay by RT-PCR confirmed IRF-7 mRNA (**B**) and IL-8 mRNA (**C**) were increased in HAECs by treatment with anti-GRP78_aa 246–260_ autoantibodies, again compared with cells treated with control IgG. (**D**) IL-8 concentrations, measured by ELISA, were greater in supernatants of HAECs treated with anti-GRP78_aa 246–260_ Abs compared with those treated with normal IgG control.

Treatment of HAECs with anti-GRP78_aa 246–260_ altered a number of gene expressions, notably including (among other effects) increasing transcriptions of IL-8 and IRF-7 mRNA (Fig. 5A). These particular findings were further validated by quantitative RT-PCR (Fig. 5B, 5C) as well as ELISA to measure HAEC IL-8 chemokine secretion (Fig. 5D).

## DISCUSSION

These data show significant associations between anti-GRP78 IgG autoantibodies and carotid atherosclerosis in COPD patients. These relationships were strongest among males with moderate to severe expiratory airflow obstruction (Figs. 1, 2) who also happen to be the subpopulation of smokers at greatest risk for CVD. The increased risk of carotid abnormalities attributable to anti-GRP78 autoreactivity persisted despite adjustment for age, smoking extent, hypertension, diabetes, hyperlipidemia, or levels of CRP, IL-6, and TNF-α (Fig. 3). Several previous reports have described linkages between autoimmunity and atherosclerosis in other disease populations and animal models (32–34). Nonetheless, to our knowledge, this is the first study that has identified an Ag-specific adaptive immune response in COPD patients that is associated with atherosclerosis.

Several additional data in this study substantiate the biological plausibility and relevance of these findings. The development of autoimmunity is dependent on the accessibility of autoantigen(s) to immune effectors. GRP78 has been shown to be expressed by various cancer cells (18), leukocytes (12), and vascular endothelial cells activated by ER stress (22). The present studies confirm GRP78 expression is increased on HAECs during ER stress, as well as by culture in media subjected to disturbed flow, which replicates the physiological shear stresses these cells are exposed to in vascular bifurcations wherein atherosclerotic plaques preferentially develop (Fig. 4) (35). The overexpression of an otherwise nonimmunogenic self-protein is a risk factor for development of autoimmune responses to that protein, particularly if the overexpression is proximate to inflammatory foci (36–39). Intrapulmonary GRP78 levels are significantly increased in a number of inflammation-associated lung injuries, including noxious smoke exposure and COPD (12, 40).

Other mechanisms may also contribute to the induction of anti-GRP78 autoimmunity in COPD. Microbial colonization and/or infections are frequent (if not ubiquitous), in smoke-damaged lungs, are highly immunogenic, and are likely a predilection for subsequent development of autoimmunity against GRP78 and other HSP by epitope spread and mimicry (9, 41, 42). Increased local concentrations of HSP, including GRP78, that are proximate to injury foci may also promote “bystander” autoreactivity because of the close physical associations of these carrier molecules with foreign (e.g., microbial) Ags or other immunogenic proteins (43, 44). Covalent modifications of immunologically inert autologous proteins by highly reactive constituents of inhaled tobacco smoke can also result in generation of “neoantigens” that are no longer recognized as self (45, 46).

The data in this study also extend previous reports that have shown anti-GRP78 Abs can deleteriously alter functions of target cells, presumably by cross-linking the GRP78 bound to surface receptors (17, 20, 21, 31, 36). We recently found circulating anti-GRP78 IgG isolated from humans with COPD binds to monocyte lineage cells and increases NF-κB phosphorylation and productions of IL-8, CCL2 (MCP-1), and MMP9 among those leukocytes (12). Another report showed autoantibodies raised in *ApoE*^−/−^ mice by immunization with rGRP78 can induce expression of adhesion molecules (22). The present study shows that actual human in situ anti-GRP78_aa 246–260_ IgG autoantibodies, isolated directly from the plasma of COPD patients, are not only highly associated with cIMT (Fig. 2), but also have biologically relevant effects on vascular endothelial cells, which notably include inducing these cells to increase their productions of IL-8 and IRF-7 (Fig. 5). IL-8 causes rolling leukocytes to adhere to endothelial cells, is mitogenic and chemotactic for vascular smooth muscle cells, and is increased in unstable atherosclerotic plaques (47). IRF-7 acts as an integrative stress sensor that mediates signaling from pattern recognition receptors via MyD88 and, among other downstream effects, promotes the transcription of proatherosclerotic type I IFN (48).

To our knowledge, another novel finding of the current report is the discovery that carotid atherosclerosis in COPD patients seems to be especially associated with anti-GRP78 autoantibodies that have specificity for a distinct, internal GRP78 epitope (Fig. 2). The biological effects of various anti-GRP78 Abs seem to differ considerably, depending on the particular site of their binding: some anti-GRP78 autoantibodies stimulate certain target cell functions, whereas others that bind different regions of GRP78 are inhibitory (17, 31, 49). The present findings raise hopes that otheranti-GRP78 autoantibodies could ameliorate or even counter deleterious effects of anti-GRP78_aa 246–260_, and a search for what could be “antiatherogenic” autoantibodies is ongoing. Incremental studies will also determine the clinical importance of cellular autoimmunity to GRP78_aa 246–260_ by testing the antigenicity of this peptide among T cells from COPD patients with and without cIMT abnormalities (12). Moreover, identification and subsequent use of the most clinically relevant linear peptide Ags in ELISA could obviate interassay variations of ligand binding because of the conformational variances of the larger, full-length rGRP78 molecule. The findings in this study that the cIMT association is stronger with anti-GRP78_aa 246–260_ (Fig. 2) than with the more complex admixture of autoantibodies against whole, intact GRP78 (Fig. 1) indicates the use of more-defined ligands will optimize the operating characteristics of these and related autoimmune assays.

Limitations of the current study include the use of a cohort that excluded subjects with recent cardiovascular events, those otherwise who would be at an increased risk because of morbid obesity, and all those with previous thoracic surgeries (e.g., coronary revascularization). Accordingly, the prevalence (and/or severity) of atherosclerosis in the subjects in this study is likely less than in unselected COPD patients. Because the operating characteristics of diagnostic assays are generally greatest in populations in which the disease prevalence (or severity) is highest (50), it is possible the clinical-immunological associations found in this study (Figs. 1, 2) may be even stronger in a general, unselected COPD population. Our vascular assessments were limited to carotid arteries, but the present findings almost certainly have general relevance for CVD in this patient population because carotid plaque assessment by ultrasound is a valid surrogate of coronary and other atherosclerotic abnormalities (23). We also did not thoroughly test for autoantibodies against conformational GRP78 epitopes, given the technical complexity of such studies, and those other anti-GRP78 Igs could additionally contribute to vascular disease.

Furthermore, the reductionist approach we employed in the current study to characterize unique characteristics of the anti-GRP78 IgG autoantibodies in COPD patients almost certainly underestimated the aggregate pathologic effects of these autoimmune responses in vivo. IgG autoantibodies are capable of forming complexes with Ag and activating complement, and these highly injurious processes, which also appear to be operative in COPD (9–12, 30), result in neutrophil recruitment to inflammatory foci and cytotoxicity of target cells (30). IgG binding to Fc receptors on granulocytes, phagocytes, NK cells, and B cells (among other subpopulations) can transduce signals that activate or otherwise deleteriously alter cell functions (29). Accordingly, we employed concurrent treatments with normal IgG as comparators to parse out effects of the anti-GRP78 IgG that are singularly attributable to their cognate specificity. Almost certainly, however, nonspecific binding of anti-GRP78 autoantibodies to cellular Fc receptors also has disease-relevant consequence in COPD patients. Moreover, autoantibody production against peptide autoantigens is typically dependent on help from T cells that share common Ag specificity, and the anti-GRP78 T cells that are present in COPD patients are also likely pathogenic (12) but were not examined in this study. Atherosclerosis is a complex syndrome and, as such, other distinct immune mechanisms and autoimmune responses may also play roles.

Better understandings of the immunopathogenic processes that can cause or contribute to atherosclerosis would have several important ramifications. Facile bioassays could be devised to enable screening of large susceptible populations, as well as expedite more focused and/or frequent clinical surveillance of those individuals at greatest risk. Anti-GRP78_aa 246–260_ ELISA ODs are already employed as the surrogate end point in an early phase clinical study (NCT03244059). Future uses could also include optimizing the selection of high-risk participants in expensive long-term intervention trials. Perhaps most importantly, many autoimmune diseases are, like COPD, not especially amenable to nonspecific immunosuppressive treatments commonly used in these lung disease patients. In contrast, regimens that target the underlying immunological mechanism(s) (e.g., autoantibodies) are more often beneficial (51–53). The present data, and related reports, provide reason to believe that innovative therapies, focused at one or more critical elements of the causal autoimmune cascade(s), might have unprecedented efficacy for prevention or treatment of these morbid disorders.

## Supplementary Material

supplement

## Figures and Tables

**TABLE I.** Characteristics of the study participants

	SC	COPD	*p* Value
*N*	81	63	
Age (years)	62.3 ± 4.2	65.9 ± 6.1	0.0002
Gender (male, %)	48.1	52.4	NS
Smoking exposure (pack years, %)	39.8 ± 15.6	54.5 ± 24.8	0.0002
Current smokers (%)	40.7	39.7	NS
FEV1 (pp values)	97.1 ± 10.3	71.1 ± 17.8	<0.0001
FEV1/FVC	0.78 ± 0.04	0.60 ± 0.11	<0.0001
DLCO (pp values)	84.0 ± 0.12	67.9 ± 16.7	<0.0001
F_950_	0.006 ± 0.007	0.052 ± 0.081	<0.0001
Anti-GRP78 (full-length) (OD)	1.11 ± 0.42	1.11 ± 0.39	NS
Anti-GRP78_aa 246–260_ (OD)	0.31 ± 0.46	0.27 ± 0.08	0.03
cIMT (cm)	0.90 ± 0.17	0.89 ± 0.15	NS
Percentage with abnormal cIMT (>0.90 mm)	49.4	38.1	NS
Hypertension (%)	57.5	65.1	NS
Diabetes (%)	11.2	9.5	NS
Hyperlipidemia (%)	58.8	54.0	NS
Percentage with at least one risk factor	76.5	82.5	NS
hs-CRP (μg/ml)	1.5 ± 2.1	3.1 ± 3.2	0.0005
IL-6 (pg/ml)	1.3 ± 0.8	2.3 ± 2.5	NS
TNF-α (pg/ml)	2.4 ± 1.3	2.2 ± 1.2	NS

DLCO, diffusing capacity of the lungs for carbon monoxide (a correlate of intrapulmonary gas exchange); F_950_, percentages of lung voxels ≤–950 Hounsfield units on inspiratory chest CT scans.

## Abbreviations used in this article:

CI: confidence interval
cIMT: carotid intima-medial thickness
COPD: chronic obstructive pulmonary disease
CT: computed tomography
CVD: cardiovascular disease
ECCM: endothelial cell culture model
ER: endoplasmic reticulum
FEV1: forced expiratory volume in 1 s
FVC: forced vital capacity
GOLD: Global Initiative for Chronic Obstructive Lung Disease
GRP78: glucose-regulated protein 78
HAEC: human aortic endothelial cell
hs-CRP: high-sensitivity CRP
HSP: heat shock protein
IRF-7: IFN regulatory factor-7
OR: odds ratio
pp: percent predicted
rGRP78: recombinant GRP78
SC: smoke control
UPR: unfolded protein response

## References

[1] Xu J, Murphy SL, Kochanek KD, Bastian B, and Arias E. 2018 Deaths: final data for 2016. National Vital Statistics Reports 67: 1–76. 30248015

[2] McGarvey LP, John M, Anderson JA, Zvarich M, and Wise RA; TORCH Clinical Endpoint Committee. 2007 Ascertainment of cause-specific mortality in COPD: operations of the TORCH Clinical Endpoint Committee. Thorax 62: 411–415. 17311843 10.1136/thx.2006.072348 PMC2117197

[3] Anthonisen NR, Connett JE, Enright PL, and Manfreda J; Lung Health Study Research Group. 2002 Hospitalizations and mortality in the lung health study. Am. J. Respir. Crit. Care Med 166: 333–339. 12153966 10.1164/rccm.2110093

[4] Roversi S, Fabbri LM, Sin DD, Hawkins NM, and Agustí A. 2016 Chronic obstructive pulmonary disease and cardiac diseases. An urgent need for integrated care. Am. J. Respir. Crit. Care Med 194: 1319–1336. 27589227 10.1164/rccm.201604-0690SO

[5] Lahousse L, Tiemeier H, Ikram MA, and Brusselle GG. 2015 Chronic obstructive pulmonary disease and cerebrovascular disease: a comprehensive review. Respir. Med 109: 1371–1380. 26342840 10.1016/j.rmed.2015.07.014

[6] Sin DD, Wu L, and Man SF. 2005 The relationship between reduced lung function and cardiovascular mortality: a population-based study and a systematic review of the literature. Chest 127: 1952–1959. 15947307 10.1378/chest.127.6.1952

[7] Sorlie PD, Kannel WB, and O’Connor G. 1989 Mortality associated with respiratory function and symptoms in advanced age. The Framingham Study. Am. Rev. Respir. Dis 140: 379–384. 2764375 10.1164/ajrccm/140.2.379

[8] Tamagawa E, and van Eeden SF. 2006 Impaired lung function and risk for stroke: role of the systemic inflammation response? Chest 130: 1631–1633. 17166971 10.1378/chest.130.6.1631

[9] Feghali-Bostwick CA, Gadgil AS, Otterbein LE, Pilewski JM, Stoner MW, Csizmadia E, Zhang Y, Sciurba FC, and Duncan SR. 2008 Autoantibodies in patients with chronic obstructive pulmonary disease. Am. J. Respir. Crit. Care Med 177: 156–163. 17975205 10.1164/rccm.200701-014OC PMC2204079

[10] Núñez B, Sauleda J, Antó JM,´ Julià MR, Orozco M, Noguera E. Monsó, Gómez FP, Garcia-Aymerich J, and Agustí A, PAC-COPD Investigators. 2011 Anti-tissue antibodies are related to lung function in chronic obstructive pulmonary disease. Am. J. Respir. Crit. Care Med 183: 1025–1031. 21097696 10.1164/rccm.201001-0029OC

[11] Packard TA, Li QZ, Cosgrove GP, Bowler RP, and Cambier JC. 2013 COPD is associated with production of autoantibodies to a broad spectrum of self-antigens, correlative with disease phenotype. Immunol. Res 55: 48–57. 22941590 10.1007/s12026-012-8347-x PMC3919062

[12] Bon J, Kahloon R, Zhang Y, Xue J, Fuhrman CR, Tan J, Burger M, Kass DJ, Csizmadia E, Otterbein L, 2014 Autoreactivity to glucose regulated protein 78 links emphysema and osteoporosis in smokers. PLoS One 9: e105066. 25216103 10.1371/journal.pone.0105066 PMC4162538

[13] Browning JL 2006 B cells move to centre stage: novel opportunities for autoimmune disease treatment. Nat. Rev. Drug Discov 5: 564–576. 16816838 10.1038/nrd2085

[14] Curtis JL, Freeman CM, and Hogg JC. 2007 The immunopathogenesis of chronic obstructive pulmonary disease: insights from recent research. Proc. Am. Thorac. Soc 4: 512–521. 17878463 10.1513/pats.200701-002FM PMC2365762

[15] Vuga LJ, Tedrow JR, Pandit KV, Tan J, Kass DJ, Xue J, Chandra D, Leader JK, Gibson KF, Kaminski N, 2014 C-X-C motif chemokine 13 (CXCL13) is a prognostic biomarker of idiopathic pulmonary fibrosis. Am. J. Respir. Crit. Care Med 189: 966–974. 24628285 10.1164/rccm.201309-1592OC PMC4098096

[16] Xue J, Kass DJ, Bon J, Vuga L, Tan J, Csizmadia E, Otterbein L, Soejima M, Levesque MC, Gibson KF, 2013 Plasma B lymphocyte stimulator and B cell differentiation in idiopathic pulmonary fibrosis patients. J. Immunol 191: 2089–2095. 23872052 10.4049/jimmunol.1203476 PMC3804013

[17] Gonzalez-Gronow M, Selim MA, Papalas J, and Pizzo SV. 2009 GRP78: a multifunctional receptor on the cell surface. Antioxid. Redox Signal 11: 2299–2306. 19331544 10.1089/ARS.2009.2568

[18] Lee AS 2014 Glucose-regulated proteins in cancer: molecular mechanisms and therapeutic potential. Nat. Rev. Cancer 14: 263–276. 24658275 10.1038/nrc3701 PMC4158750

[19] Blumental-Perry A 2012 Unfolded protein response in chronic obstructive pulmonary disease: smoking, aging and disease: a SAD trifecta. Curr. Mol. Med 12: 883–898. 22697343 10.2174/156652412801318764

[20] Liu R, Li X, Gao W, Zhou Y, Wey S, Mitra SK, Krasnoperov V, Dong D, Liu S, Li D, 2013 Monoclonal antibody against cell surface GRP78 as a novel agent in suppressing PI3K/AKT signaling, tumor growth, and metastasis. Clin. Cancer Res 19: 6802–6811. 24048331 10.1158/1078-0432.CCR-13-1106 PMC4151476

[21] Misra UK, and Pizzo SV. 2010 Ligation of cell surface GRP78 with antibody directed against the COOH-terminal domain of GRP78 suppresses Ras/MAPK and PI 3-kinase/AKT signaling while promoting caspase activation in human prostate cancer cells. Cancer Biol. Ther 9: 142–152. 20368692 10.4161/cbt.9.2.10422

[22] Crane ED, Al-Hashimi AA, Chen J, Lynn EG, Won KD, Lhotàk Š, Naeim M, Platko K, Lebeau P, Byun JH, 2018 Anti-GRP78 autoantibodies induce endothelial cell activation and accelerate the development of atherosclerotic lesions. JCI Insight 3: e99363. 10.1172/jci.insight.99363 PMC6338388 30568038

[23] Nezu T, Hosomi N, Aoki S, and Matsumoto M. 2016 Carotid intima-media thickness for atherosclerosis. J. Atheroscler. Thromb 23: 18–31. 26460381 10.5551/jat.31989

[24] Global Initiative for Chronic Obstructive Lung Disease. 2018 Global strategy for diagnosis, management, and prevention of COPD. Available at: https://goldcopd.org/wp-content/uploads/2017/11/GOLD-2018-v6.0-FINAL-revised-20-Nov_WMS.pdf.

[25] Bon J, Fuhrman CR, Weissfeld JL, Duncan SR, Branch RA, Chang CC, Zhang Y, Leader JK, Gur D, Greenspan SL, and Sciurba FC. 2011 Radiographic emphysema predicts low bone mineral density in a tobacco-exposed cohort. Am. J. Respir. Crit. Care Med 183: 885–890. 20935108 10.1164/rccm.201004-0666OC PMC3086755

[26] Barr RG, Berkowitz EA, Bigazzi F, Bode F, Bon J, Bowler RP, Chiles C, Crapo JD, Criner GJ, Curtis JL, ; COPDGene CT Workshop Group. 2012 A combined pulmonary-radiology workshop for visual evaluation of COPD: study design, chest CT findings and concordance with quantitative evaluation. COPD 9: 151–159. 22429093 10.3109/15412555.2012.654923 PMC3752926

[27] Patibandla PK, Rogers AJ, Giridharan GA, Pallero MA, Murphy-Ullrich JE, and Sethu P. 2014 Hyperglycemic arterial disturbed flow niche as an in vitro model of atherosclerosis. Anal. Chem 86: 10948–10954. 25279658 10.1021/ac503294p

[28] Livak KJ, and Schmittgen TD. 2001 Analysis of relative gene expression data using real-time quantitative PCR and the 2(-Delta C(T)) method. Methods 25: 402–408. 11846609 10.1006/meth.2001.1262

[29] Forthal DN 2014 Functions of antibodies. Microbiol. Spectr 2: 1–17. PMC4159104 25215264

[30] Mayadas TN, Tsokos GC, and Tsuboi N. 2009 Mechanisms of immune complex-mediated neutrophil recruitment and tissue injury. Circulation 120: 2012–2024. 19917895 10.1161/CIRCULATIONAHA.108.771170 PMC2782878

[31] Gonzalez-Gronow M, Cuchacovich M, Llanos C, Urzua C, Gawdi G, and Pizzo SV. 2006 Prostate cancer cell proliferation in vitro is modulated by antibodies against glucose-regulated protein 78 isolated from patient serum. Cancer Res. 66: 11424–11431. 17145889 10.1158/0008-5472.CAN-06-1721

[32] Foteinos G, Afzal AR, Mandal K, Jahangiri M, and Xu Q. 2005 Anti-heat shock protein 60 autoantibodies induce atherosclerosis in apolipoprotein E-deficient mice via endothelial damage. Circulation 112: 1206–1213. 16116071 10.1161/CIRCULATIONAHA.105.547414

[33] Hsue PY, Scherzer R, Grunfeld C, Imboden J, Wu Y, Del Puerto G, Nitta E, Shigenaga J, Schnell Heringer A, Ganz P, and Graf J. 2014 Depletion of B-cells with rituximab improves endothelial function and reduces inflammation among individuals with rheumatoid arthritis. J. Am. Heart Assoc 3: e001267. 25336464 10.1161/JAHA.114.001267 PMC4323827

[34] Bugała K, Mazurek A, Gryga K, Komar M, Kopeć G, Musiał J, Podolec P, Perricone C, and Płazak W. 2018 Influence of autoimmunity and inflammation on endothelial function and thrombosis in systemic lupus erythematosus patients. Clin. Rheumatol 37: 2087–2093. 29675623 10.1007/s10067-018-4104-4

[35] Estrada R, Giridharan GA, Nguyen MD, Prabhu SD, and Sethu P. 2011 Microfluidic endothelial cell culture model to replicate disturbed flow conditions seen in atherosclerosis susceptible regions. Biomicrofluidics 5: 32006–3200611. 22662029 10.1063/1.3608137 PMC3364817

[36] Kurts C, Sutherland RM, Davey G, Li M, Lew AM, Blanas E, Carbone FR, Miller JF, and Heath WR. 1999 CD8 T cell ignorance or tolerance to islet antigens depends on antigen dose. Proc. Natl. Acad. Sci. USA 96: 12703–12707. 10535986 10.1073/pnas.96.22.12703 PMC23058

[37] Rush C, Mitchell T, and Garside P. 2002 Efficient priming of CD4+ and CD8+ T cells by DNA vaccination depends on appropriate targeting of sufficient levels of immunologically relevant antigen to appropriate processing pathways. J. Immunol 169: 4951–4960. 12391208 10.4049/jimmunol.169.9.4951

[38] Martinic MM, Huber C, Coppieters K, Oldham JE, Gavin AL, and von Herrath MG. 2010 Expression level of a pancreatic neo-antigen in beta cells determines degree of diabetes pathogenesis. J. Autoimmun 35: 404–413. 20932718 10.1016/j.jaut.2010.08.006 PMC2963714

[39] George TC, Bilsborough J, Viney JL, and Norment AM. 2003 High antigen dose and activated dendritic cells enable Th cells to escape regulatory T cell-mediated suppression in vitro. Eur. J. Immunol 33: 502–511. 12645949 10.1002/immu.200310026

[40] Aksoy MO, Kim V, Cornwell WD, Rogers TJ, Kosmider B, Bahmed K, Barrero C, Merali S, Shetty N, and Kelsen SG. 2017 Secretion of the endoplasmic reticulum stress protein, GRP78, into the BALF is increased in cigarette smokers. Respir. Res 18: 78. 28464871 10.1186/s12931-017-0561-6 PMC5414124

[41] Cusick MF, Libbey JE, and Fujinami RS. 2012 Molecular mimicry as a mechanism of autoimmune disease. Clin. Rev. Allergy Immunol 42: 102–111. 22095454 10.1007/s12016-011-8294-7 PMC3266166

[42] Rodríguez-Iturbe B, and Johnson RJ. 2018 Heat shock proteins and cardiovascular disease. Physiol. Int 105: 19–37. 29602292 10.1556/2060.105.2018.1.4

[43] Srivastava P 2002 Interaction of heat shock proteins with peptides and antigen presenting cells: chaperoning of the innate and adaptive immune responses. Annu. Rev. Immunol 20: 395–425. 11861608 10.1146/annurev.immunol.20.100301.064801

[44] Rajaiah R, and Moudgil KD. 2009 Heat-shock proteins can promote as well as regulate autoimmunity. Autoimmun. Rev 8: 388–393. 19121415 10.1016/j.autrev.2008.12.004 PMC2668694

[45] Kirkham PA, and Barnes PJ. 2013 Oxidative stress in COPD. Chest 144: 266–273. 23880677 10.1378/chest.12-2664

[46] Kheradmand F, Shan M, Xu C, and Corry DB. 2012 Autoimmunity in chronic obstructive pulmonary disease: clinical and experimental evidence. *Expert Rev*. Clin. Immunol 8: 285–292. 10.1586/eci.12.7 PMC3321360 22390492

[47] Apostolakis S, Vogiatzi K, Amanatidou V, and Spandidos DA. 2009 Interleukin 8 and cardiovascular disease. Cardiovasc. Res 84: 353–360. 19617600 10.1093/cvr/cvp241

[48] Gordts PLSM, Foley EM, Lawrence R, Sinha R, Lameda-Diaz C, Deng L, Nock R, Glass CK, Erbilgin A, Lusis AJ, 2014 Reducing macrophage proteoglycan sulfation increases atherosclerosis and obesity through enhanced type I interferon signaling. Cell Metab. 20: 813–826. 25440058 10.1016/j.cmet.2014.09.016 PMC4254584

[49] Misra UK, and Pizzo SV. 2010 Modulation of the unfolded protein response in prostate cancer cells by antibody-directed against the carboxyl-terminal domain of GRP78. Apoptosis 15: 173–182. 20091233 10.1007/s10495-009-0430-y

[50] Hajian-Tilaki K 2013 Receiver operating characteristic (ROC) curve analysis for medical diagnostic test evaluation. Caspian J. Intern. Med 4: 627–635. 24009950 PMC3755824

[51] Erickson SB, Kurtz SB, Donadio JV Jr., Holley KE, Wilson CB, and Pineda AA. 1979 Use of combined plasmapheresis and immunosuppression in the treatment of Goodpasture’s syndrome. Mayo Clin. Proc 54: 714–720. 491763

[52] Sem M, Molberg O, Lund MB, and Gran JT. 2009 Rituximab treatment of the anti-synthetase syndrome: a retrospective case series. Rheumatology (Oxford) 48: 968–971. 19531628 10.1093/rheumatology/kep157

[53] Gelfand EW 2012 Intravenous immune globulin in autoimmune and inflammatory diseases. N. Engl. J. Med 367: 2015–2025. 23171098 10.1056/NEJMra1009433

