# Supplementary material for: Glucose-Regulated Protein 78 Autoantibodies Are Associated with Carotid Atherosclerosis in Chronic Obstructive Pulmonary Disease Patients
Source: Immunohorizons. Author manuscript; Available in PMC 2020 Aug 17. (PMC7430561; doi:10.4049/immunohorizons.1900098)
Supplement: supplement [file NIHMS1617340-supplement-supplement.pdf]

[1] **Supplement Table I.** GRP78 peptides synthesized for individual use as ligands in ELISA

	aa location	sequence
1	17-30	Biot-Ahx-RAEEEDKKEDVGTV-amide
2	64-81	Biot-Ahx-SYVAFTPEGERLIGDAAK-amide
3	78-93	Biot-Ahx-DAAKNQLTSNPENTVF-amide
4	98-115	Biot-Ahx-LIGRTWNDPSVQQDIKFL-amide
5	120-136	Biot-Ahx-VEKKTKPYIQVDIGGGQ-amide
6	133-145	Biot-Ahx-GGGQTKTFAPEEI-amide
7	154-162	Biot-Ahx-KETAEAYLG-amide
8	200-218	Biot-Ahx-NEPTAAAIAYGLDKREGEK-amide
9	246-260	Biot-Ahx-ATNGDTHLGGEDFDQ-amide
10	272-285	Biot-Ahx-KKTGKDVRKDNRAV-amide
11	336-356	Biot-Ahx-RSTMKPVQKVLEDSDLKKSDI-amide
12	364-371	Biot-Ahx-GSTRIPKI-amide
13	379-398	Biot-Ahx-FNGKEPSRGINPDEAVAYGA-amide
14	395-413	Biot-Ahx-AYGAAVQAGVLSGDQDTGD-amide
15	466-477	Biot-Ahx-YEGERPLTKDNH-amide
16	484-497	Biot-Ahx-LTGIPPAPRGVPQI-amide
17	512-530	Biot-Ahx-TAEDKGTGNKNKITITNDQ-amide
18	527-542	Biot-Ahx-TNDQNRLTPEEIERMV-amide
19	539-557	Biot-Ahx-ERMVNDAEKFAEEDKKLKE-amide
20	553-367	Biot-Ahx-KKLERIDTRNELES-amide
21	573-591	Biot-Ahx-KNQIGDKEKLGGKLSSEDK-amide
22	587-600	Biot-Ahx-SSEDKETMEKAVEE-amide
23	605-625	Biot-Ahx-LESHQDADIEDFKAKKKELEE-amide
24	635-654	Biot-Ahx-YGSAGPPPTGEEDTAEKDEL-amide

[1] These sequences were identified as potential linear epitopes of GRP78 using the BepiPhred-2.0 sequential B cell-epitope predictor (http://www.cbs.dtu.dk/services/ BepiPred/).

Biot = biotin; Ahx = aminohexanoic linker

2 **Supplement Table II.** GRP78 peptide ELISA results

	**aa location**	**sequence**	***r*s**	**p-value**
1	17-30	Biot-Ahx-RAEEEDKKEDVGTV-amide	-0.28	0.18
2	64-81	Biot-Ahx-SYVAFTPEGERLIGDAAK-amide	-0.08	0.69
3	78-93	Biot-Ahx-DAAKNQLTSNPENTVF-amide	-0.12	0.55
4	98-115	Biot-Ahx-LIGRTWNDPSVQQDIKFL-amide	0.08	0.31
5	120-136	Biot-Ahx-VEKKTKPYIQVDIGGGQ-amide	0.00	1.00
6	133-145	Biot-Ahx-GGGQTKTFAPEEI-amide	-0.01	0.97
7	154-162	Biot-Ahx-KETAEAYLG-amide	-0.10	0.64
8	200-218	Biot-Ahx-NEPTAAAIAYGLDKREGEK-amide	0.24	0.24
9	**246-260**	**Biot-Ahx-ATNGDTHLGGEDFDQ-amide**	**0.23**	**0.01**
10	272-285	Biot-Ahx-KKTGKDVRKDNRAV-amide	0.02	0.80
11	336-356	Biot-Ahx-RSTMKPVQKVLEDSDLKKSDI-amide	0.15	0.08
12	364-371	Biot-Ahx-GSTRIPKI-amide	0.19	0.03
13	379-398	Biot-Ahx-FNGKEPSRGINPDEAVAYGA-amide	0.08	0.41
14	395-413	Biot-Ahx-AYGAAVQAGVLSGDQDTGD-amide	-0.06	0.77
15	466-477	Biot-Ahx-YEGERPLTKDNH-amide	-0.01	0.95
16	484-497	Biot-Ahx-LTGIPPAPRGVPQI-amide	-0.04	0.84
17	512-530	Biot-Ahx-TAEDKGTGNKNKITITNDQ-amide	-0.13	0.52
18	527-542	Biot-Ahx-TNDQNRLTPEEIERMV-amide	-0.01	0.96
19	539-557	Biot-Ahx-ERMVNDAEKFAEEDKKLKE-amide	0.19	0.32
20	553-367	Biot-Ahx-KKLERIDTRNELES-amide	-0.10	0.64
21	573-591	Biot-Ahx-KNQIGDKEKLGGKLSSEDK-amide	0.00	1.00
22	587-600	Biot-Ahx-SSEDKETMEKAVEE-amide	0.09	0.30
23	605-625	Biot-Ahx-LESHQDADIEDFKAKKKELEE-amide	0.11	0.59
24	635-654	Biot-Ahx-YGSAGPPPTGEEDTAEKDEL-amide	0.08	0.55

2 Results of ELISAs using individual synthesized GRP78 peptides as ligands in assays of all plasma specimens within the participant cohort. Shown are Spearman rank correlations (r_s) and the corresponding p values of the ELISA associations with carotid intima thickness (cIMT).

Peptide #9 (**BOLD**) was the GRP78 epitope for autoantibodies most closely correlated with carotid intima thicknesses (cIMT).

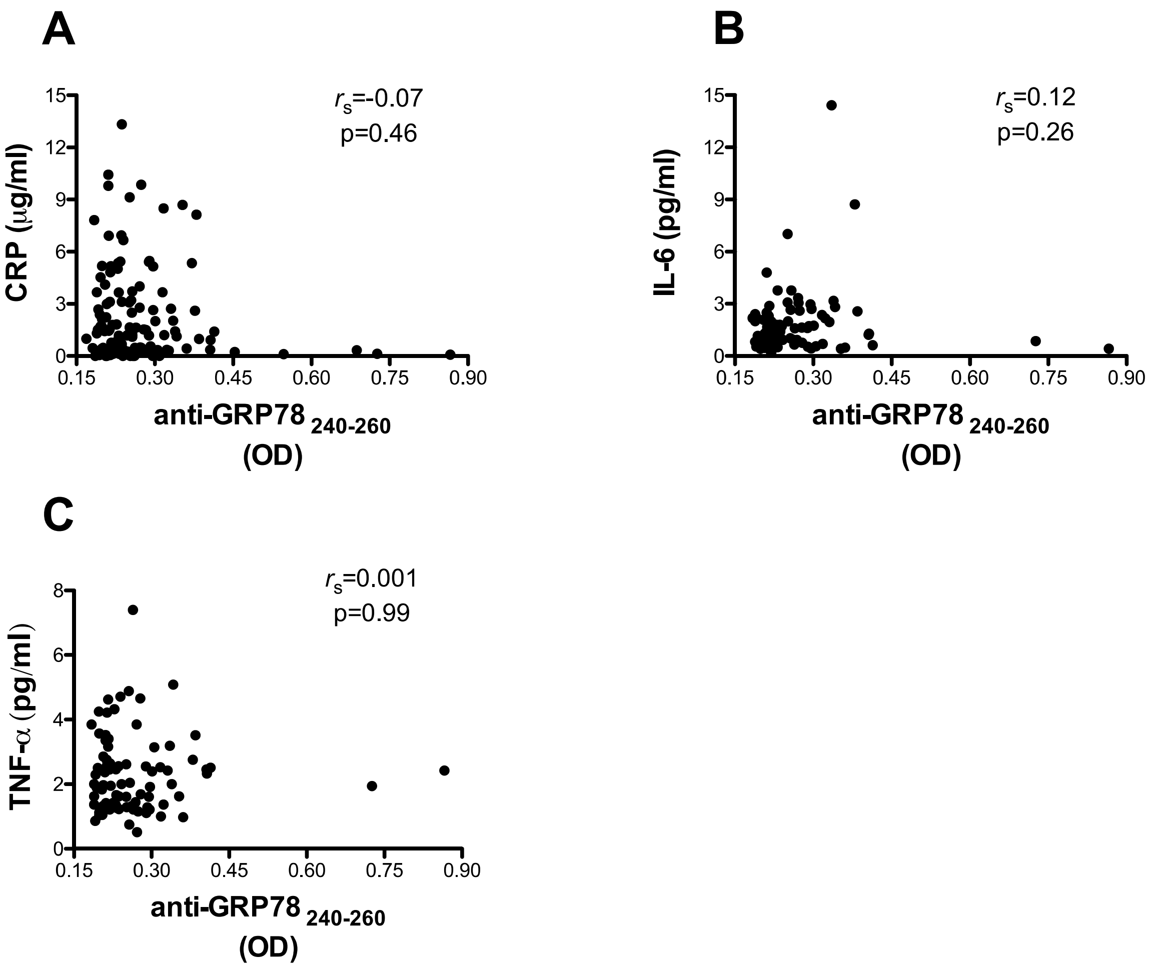

Supplemental Figure 1. Anti-GRP78 $_{\text{a.a.246-260}}$ autoantibody levels were not correlated with nonspecific inflammatory markers. No significant correlations were observed between circulating anti-GRP78 $_{\text{a.a.246-260}}$ autoantibody concentrations, as measured by optical density (OD), and concentrations of inflammatory mediators: (A) C-reactive protein (CRP); (B) Interleukin-6 (IL-6); and (C) Tumor necrosis factor-α (TNF-α).
